# Selection and Misclassification Biases in Longitudinal Studies

**DOI:** 10.3389/fvets.2018.00099

**Published:** 2018-05-28

**Authors:** Denis Haine, Ian Dohoo, Simon Dufour

**Affiliations:** ^1^Faculté de médecine vétérinaire, Université de Montréal, Montreal, QC, Canada; ^2^Canadian Bovine Mastitis and Milk Quality Research Network, St-Hyacinthe, QC, Canada; ^3^Centre for Veterinary Epidemiological Research, Atlantic Veterinary College, University of Prince Edward Island, Charlottetown, PE, Canada

**Keywords:** bias (epidemiology), longitudinal study, selection bias, misclassification, epidemiologic methods

## Abstract

Using imperfect tests may lead to biased estimates of disease frequency and measures of association. Many studies have looked into the effect of misclassification on statistical inferences. These evaluations were either within a cross-sectional study framework, assessing biased prevalence, or for cohort study designs, evaluating biased incidence rate or risk ratio estimates based on misclassification at one of the two time-points (initial assessment or follow-up). However, both observations at risk and incident cases can be wrongly identified in longitudinal studies, leading to selection and misclassification biases, respectively. The objective of this paper was to evaluate the relative impact of selection and misclassification biases resulting from misclassification, together, on measures of incidence and risk ratio. To investigate impact on measure of disease frequency, data sets from a hypothetical cohort study with two samples collected one month apart were simulated and analyzed based on specific test and disease characteristics, with no elimination of disease during the sampling interval or clustering of observations. Direction and magnitude of bias due to selection, misclassification, and total bias was assessed for diagnostic test sensitivity and specificity ranging from 0.7 to 1.0 and 0.8 to 1.0, respectively, and for specific disease contexts, i.e., disease prevalences of 5 and 20%, and disease incidences of 0.01, 0.05, and 0.1 cases/animal-month. A hypothetical exposure with known strength of association was also generated. A total of 1,000 cohort studies of 1,000 observations each were simulated for these six disease contexts where the same diagnostic test was used to identify observations at risk at beginning of the cohort and incident cases at its end. Our results indicated that the departure of the estimates of disease incidence and risk ratio from their true value were mainly a function of test specificity, and disease prevalence and incidence. The combination of the two biases, at baseline and follow-up, revealed the importance of a good to excellent specificity relative to sensitivity for the diagnostic test. Small divergence from perfect specificity extended quickly to disease incidence over-estimation as true prevalence increased and true incidence decreased. A highly sensitive test to exclude diseased subjects at baseline was of less importance to minimize bias than using a highly specific one at baseline. Near perfect diagnostic test attributes were even more important to obtain a measure of association close to the true risk ratio, according to specific disease characteristics, especially its prevalence. Low prevalent and high incident disease lead to minimal bias if disease is diagnosed with high sensitivity and close to perfect specificity at baseline and follow-up. For more prevalent diseases we observed large risk ratio biases towards the null value, even with near perfect diagnosis.

## Introduction

A cohort study is a longitudinal observational study in which a study population (i.e., a cohort) is selected and followed up in time (1, 2). Members of the cohort share a common experience (e.g., Kennel Club registered Labrador Retrievers born after January 1, 2010 (3) or condition [e.g., litters from *A. pleuropneumoniae* infected sows (4)]. Two cohorts are often included in these longitudinal studies, one experiencing a putative causal event or condition (exposed cohort), and the other being an unexposed (reference) cohort. Cohort study is the standard study design to estimate the incidence of diseases and identify their natural history, by analyzing the association between a baseline exposure and risk of disease over the follow-up period. This type of study is characterized by the identification of a disease-free population (i.e., subjects with the outcome at baseline are excluded from the follow-up), and their exposure to a risk factor is assessed. The frequency of the outcome (generally the incidence of a disease or death) is measured and related to exposure status, expressed as a risk ratio (RR). Therefore it is assumed that prevalent and non-prevalent cases can be differentiated with no error so that only susceptible individuals are included in the cohort. Incident cases are likewise supposed to be correctly identified.

However, any measurement is prone to potential errors, as a result of subjective evaluations, imperfect diagnostic tests, reporting errors (deliberate or not), recall deficiencies, or clerical errors. Obtaining “error-free” measurements is a desirable objective but it is usually much more expensive to use “gold-standard” measurements, or they are simply not available, leaving the researcher with “less-than-ideal” measurement tools. Wrong classification at baseline and at follow-up are both misclassification biases, in the former the bias resulting from misclassification could be considered a selection bias, as the wrong (diseased) subjects are included in the cohort (2) while in the latter, it would be commonly defined as misclassification bias (5). Such errors of measurement or misclassification in exposure variables, outcomes or confounders can bias inferences drawn from the data collected, often substantially (6), or decrease the power of the study (7, 8). Many studies have looked into the effect of misclassification on statistical inferences, including biased prevalence and incidence rate estimates (6, 9) and biased relative risk estimates (10, 11). Nondifferential misclassification of disease leads in general to bias towards null in the estimated associations as well as reduced statistical efficiency (7, 10, 12). This bias depends mainly on the specificity (Sp) of the test used (12). If Sp of the test is perfect, then bias is absent (13). These evaluations were, however, either within a cross-sectional study framework, assessing biased prevalence, or for cohort study designs evaluating biased incidence rate or RR estimates but based on misclassification at only one of the two time-points (initial assessment or follow-up). However, both observations at risk and incident cases can be wrongly identified in longitudinal studies, leading to selection and misclassification biases, respectively.

The objective of this paper was to evaluate the relative impact of selection and misclassification biases resulting from misclassification, together, on measures of incidence and RR.

## Material and Methods

To investigate the impact of concomitant selection and misclassification biases on measure of disease frequency, data sets from a hypothetical cohort study with two samples collected one time unit apart were simulated and analyzed based on specific test and disease characteristics, for a stable population over the follow-up time, and with no elimination of disease or clustering of observations. Direction and magnitude of bias due to selection, misclassification, and total bias was assessed for diagnostic test sensitivity (Se) and Sp ranging from 0.7 to 1.0 (0.7, 0.75, 0.8, 0.85, 0.9, 0.95, 0.98, 0.99, 1) and 0.8 to 1.0 (0.8, 0.85, 0.9, 0.95, 0.98, 0.99, 1), respectively, and for specific disease contexts, i.e., disease prevalences of 5 and 20%, and disease incidences of 0.01, 0.05, and 0.1 cases/animal-time unit. The true case status ($S_{1}$) on first sample collection was used to identify observations at risk at the beginning of the cohort, while the second ($S_{2}$) was used to identify the true outcome. A hypothetical exposure with known strength o f association (RR ∼3.0) was also generated. For demonstration purpose, simulations were also ran with a weaker RR of ∼1.5 (see Supplementary Material). A total of 1,000 cohort studies of 1,000 observations each were simulated for these six disease contexts where the same diagnostic test was used to identify observations at risk at beginning of the cohort and incident cases at its end. On each datasets new $S_{1}$ and $S_{2}$ variables were generated by applying the scenario misclassification parameters to the $S_{1}$ and $S_{2}$ samples. Incidence and measures of association with the hypothetical exposure were then computed using first the $S_{1}$ and $S_{2}$ variables (total bias), then $S_{1}$ and $S_{2}$ (selection bias only), and finally the $S_{1}$ and $S_{2}$ variables (misclassification bias only).

Disease incidence was computed as the number of new cases at the end of the cohort divided by the number at risk at its beginning. Risk ratio was computed as the ratio of the risk of disease among observations who were exposed to the risk factor, to the risk among observations who were unexposed (2). Data sets generation and estimation procedures were realized in R (14), and simulation code is available at https://github.com/dhaine/cohortBias.

## Results

**Figure 1:**
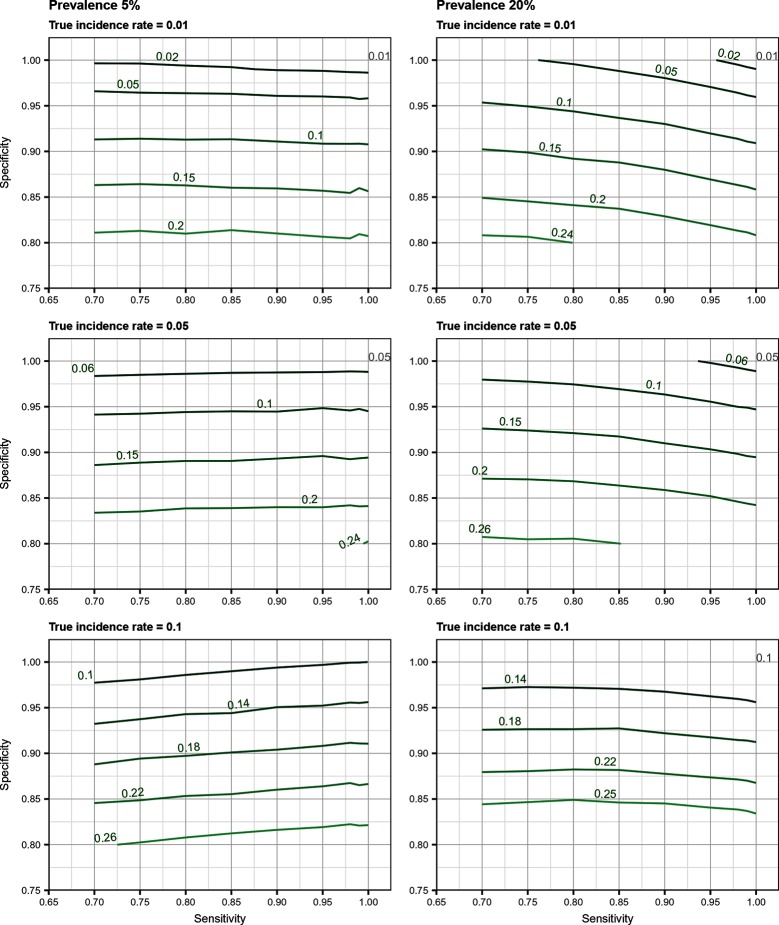
Estimated incidence rate (in cases/animal-time unit) as a function of test sensitivity and specificity, disease prevalence (5 or 20%), and true disease incidence (0.01, 0.05, 0.1 case/animal-time unit) when using an imperfect test both at baseline and follow-up (i.e., total bias). True incidence rate is found at the upper right corner (i.e., perfect sensitivity and specificity).

**Figure 2:**
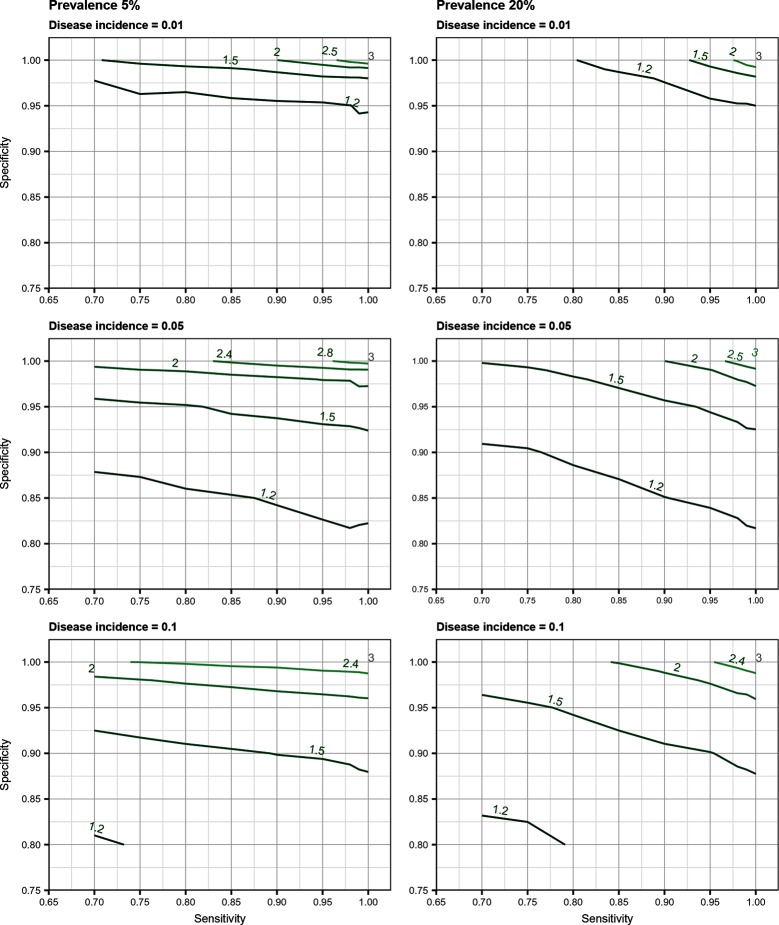
Estimated risk ratio as a function of test sensitivity and specificity, disease prevalence (5 or 20%), and true disease incidence (0.01, 0.05, 0.1 case/animal-time unit) for an exposure with a true measure of association corresponding to a risk ratio of 3.0 when using an imperfect test both at baseline and follow-up (i.e., total bias). True risk ratio is found at the upper right corner (i.e., perfect sensitivity and specificity).

**Figure 3:**
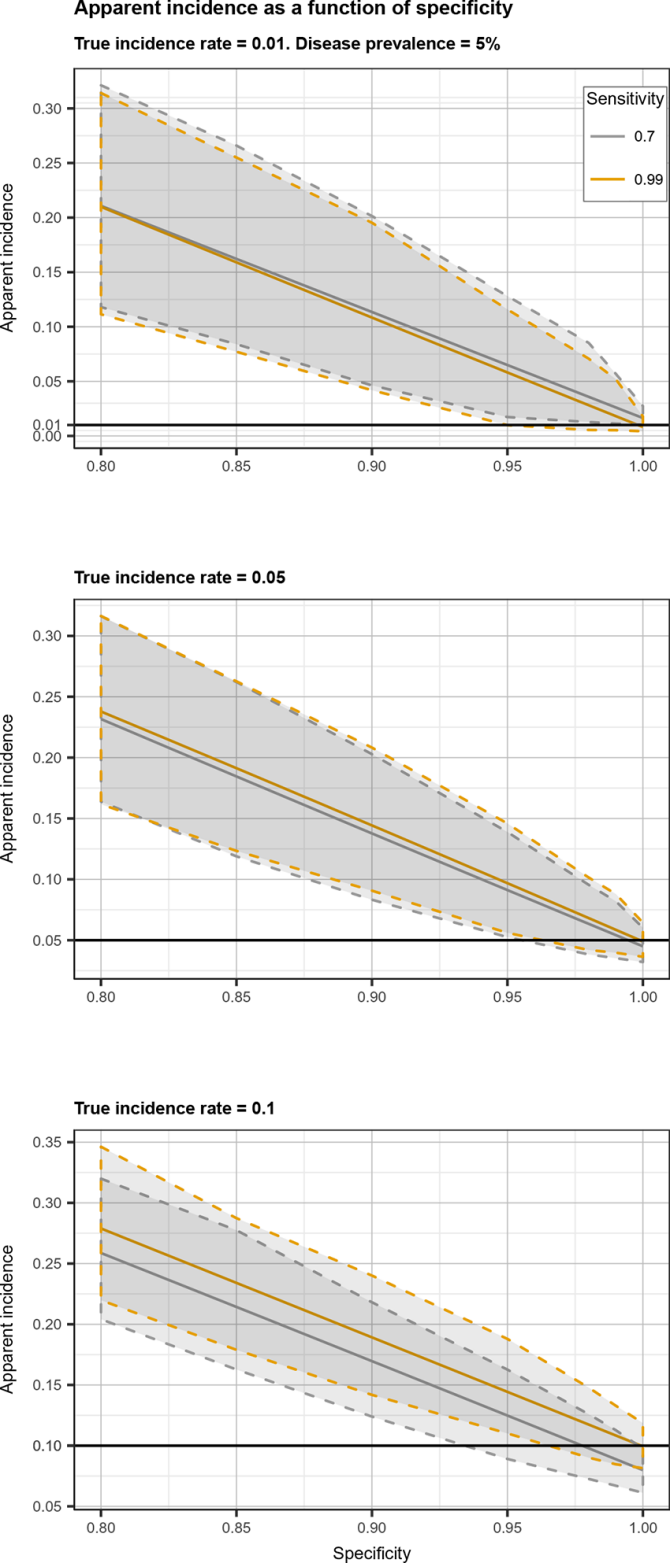
Apparent incidence resulting from total bias, as a function of specificity. Disease prevalence = 5%. Solid line: median value; dotted lines: first and third quartiles.

**Figure 4:**
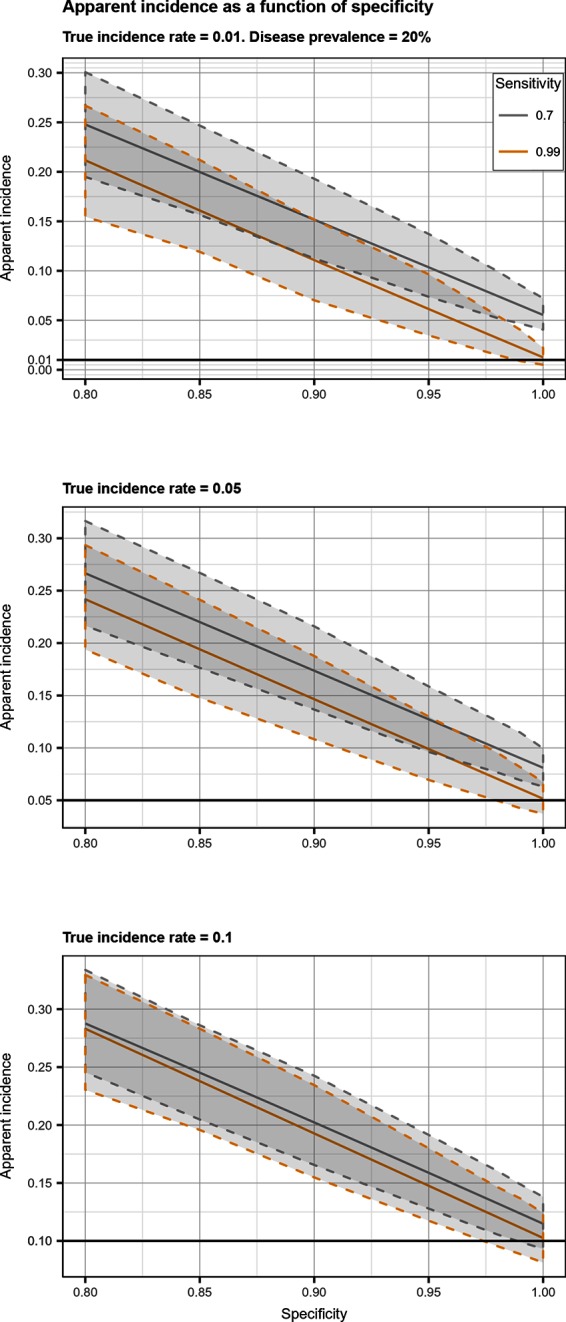
Apparent incidence resulting from total bias, as a function of specificity. Disease prevalence = 20%. Solid line: median value; dotted lines: first and third quartiles.

**Figure 5:**
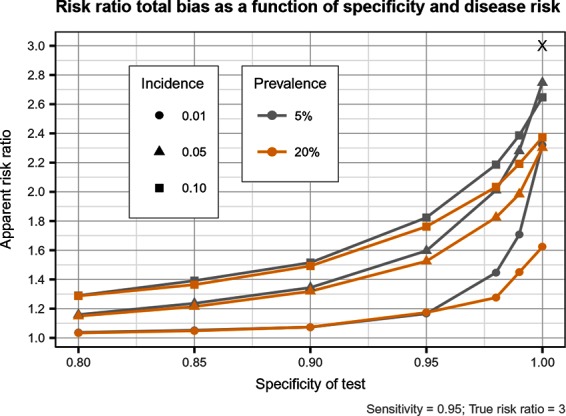
Estimated risk ratio as a function of test specificity and disease risk, and for a sensitivity of 95%, when using an imperfect test both at baseline and follow-up. True risk ratio = 3.0.

Total biases resulting from selection and misclassification errors and according to given disease prevalence, Se, and Sp are illustrated for disease incidence and RR in Figures 1, 2, respectively. These figures are contour plots where the lines are curves in the x,y-plane along which the function of the two variables on the vertical and horizontal axes (i.e., Se and Sp) has a constant value, i.e., a curve joins points of equal value (15). The true incidence rate (or RR) is therefore to be found at the upper right corner of the plot. For example, in the bottom left panel of Figure 1 the second line from the bottom is labelled 0.22. This line shows that, for a 5% disease prevalence and a true incidence rate of 0.1 case/animal-time unit, an apparent incidence estimate of 0.22 will be achieved by any combination of Sp and Se on this line (e.g., Sp = 0.845, Se = 0.7 or Sp = 0.87 and Se = 1.00). As an other example, in the upper right panel of this same figure, the first line at the top is labelled 0.02. It shows that, for a 5% disease prevalence and a true incidence rate of 0.01 case/animal-time unit, an apparent incidence estimate of 0.02 is achieved along this line by any combination of Se and Sp like, for example, a Sp of 1.00 and a Se of 0.955. The true incidence rate is given at the upper right corner, where Se and Sp are both 100%. Imperfect Se to identify individuals at risk at baseline and imperfect Sp to identify incident cases led to a mild under-estimation of the observed disease incidence (Figures S1, S2 in Supplementary Material). From these graphs we could also note that Sp has little effect on selection bias while Se has little effect on misclassification bias. Of the two, misclassification bias had a much bigger effect than selection bias. But overall, the combination of the two biases, at baseline and follow-up, revealed the importance of a good to excellent Sp relative to Se for the diagnostic test. Small divergence from perfect Sp extended quickly to disease incidence over-estimation as true prevalence increased and true incidence decreased (Figures 3–5). Selection and misclassification biases of a low prevalent and incident disease, diagnosed with close to perfect Sp, were minimal, reflecting the importance of choosing a highly specific test to improve identification of animal (or individual) unit at risk and incident case identification. The same effect was also observed with RR estimations (Figures S3, S4 in Supplementary Material). Similar results were found with a weaker exposure, RR of 1.5 (Figures S5–S8 in Supplementary Material).

## Discussion

Our results indicated that the departure of the estimates of disease incidence and risk ratio from their true value were mainly a function of test Sp, and disease prevalence and incidence. Imperfect Se to identify individuals at risk and imperfect Sp to identify incident cases led to a mild under-estimation of the observed disease incidence. The combination of the two biases, at baseline and follow-up, revealed the importance of a good to excellent Sp (over 95%) over Se for the diagnostic test. Small divergence from perfect Sp extended quickly to disease incidence over-estimation as true prevalence increased and true incidence decreased. Selection and misclassification biases of a low prevalent and incident disease, diagnosed with close to perfect Sp, were minimal, reflecting the importance of choosing a highly specific test to improve unit at risk and case identification. A highly sensitive test to exclude diseased subjects at baseline was of less importance to minimize bias than using a highly specific one at this time point. Of course, the situation would be different in a population with a very high disease prevalence. For most diseases, however, the tendency is to have a large proportion of healthy animals and a small proportion of diseased ones. The range of diseases prevalence investigated in our study (5–20%) would therefore cover most disease scenarios seen in veterinary, and perhaps, human studies.

Near perfect diagnostic test attributes were even more important to obtain a measure of association close to the true risk ratio, according to specific disease characteristics, especially its prevalence. Low prevalent and high incident disease led to minimal bias if disease was diagnosed with high Se and close to perfect Sp. For more prevalent diseases we observed large risk ratio biases towards the null value, even with near perfect diagnosis. This bias also got larger as incidence decreased. For diseases with moderate to high prevalence (20%), the biases could be so important that a study using a test with a Se or Sp <0.95 would have very little power to identify any measure of association with exposures. Even with prevalence of disease of 5%, a dramatic loss of power is to be expected when imperfect tests are used. Therefore a corollary result of a sub-optimal Sp is that, by causing a bias towards the null, weaker associations (like our RR ∼1.5) will be more difficult to demonstrate. It would be unnecessary to fight this loss in power by increasing the study sample size in order to get a narrower CI, as the measured association would be biased anyway (16). It was already demonstrated that study power decreases as misclassification increases (17). For stronger associations and in the presence of small biases, sample size could be adjusted (18, 19). But in the presence of larger biased associations towards the null, a weaker, reduced, association would be candidate for further investigation, even if its CI includes 1.0 (20).

It is already recognized that misclassification of outcome or exposure during follow-up leads to bias towards null in the estimated associations (7, 12, 21) as well as reduced statistical efficiency by loss of power (8) and confidence intervals of the parameters estimates that are too narrow (22). However this bias towards the null value is strictly true only when misclassification is the same in the two compared groups, i.e., exposure and covariates status do not influence Se and/or Sp (12, 22, 23). In this case, we have non-differential misclassification. As shown previously by (12), misclassification bias depends primarily on the Sp of the test used and increase with disease rarity, with most of the bias occurring even before the Sp drops below 85%. With Se and Sp as high as 0.90 and 0.96, respectively, RR is already substantially biased (1.5 instead of 2) (12), but when Sp is perfect, bias is absent (13). When disease frequency is low, error in disease diagnosis leads to an increase in false positives which submerge true positives and dilute measures of incidence and association. Bias in RR increases as Se increase and Sp decrease (8). Exposure misclassification alone can cause serious bias on the RR even if Se or Sp are not lower than 80% (24).

When misclassification is differential, i.e., Se and Sp of outcome classification is not equal in each true category of exposure (or Se and Sp of exposure classification is not equal in each true category of outcome), direction of bias for parameter estimates can be in any direction (22, 25, 26). In this case, Se and Sp as low as 90% can be sufficient to produce high bias (24). Direction of the bias can also be in any direction with dependent misclassification [i.e., the errors in one variable are associated with the errors in an other (27, 28)], even if non-differential (24). The same is found when the exposure variable is not dichotomous but has multiple levels (25, 29). Bias towards the null also requires that selection bias and confounding are absent (30). There are therefore many situations where bias towards null do not apply. Even when non-differential misclassification is thought to take place, random errors in the observed estimates can lead bias away from the null (30).

In cohort studies, non-differential misclassification of disease at baseline, i.e., selection bias, especially imperfect Se, can lead to over- or under-estimation of the observed RR (31). This bias can be significant for disease with a low true incidence, a high true prevalence, a substantial disease duration (i.e., as long as the interval between first and second test), and a poor test Se In the presence of misclassification of disease at baseline the observed RR depend on the association between exposure and disease both at baseline and during follow-up (31). Therefore to minimize bias, the standard recommendation is to exclude subjects with the outcome at baseline from the cohort based on a highly sensitive test (32). Then during the follow-up period, case identification should use a highly specific test having a high positive predictive value (33). However (34) have shown that a more prevalent and incident disease diagnosed with an imperfect Se and/or Sp will give biased measure of association despite attempts to improve its diagnosis.

We have shown here that combined misclassification at baseline and follow-up requires a highly specific test. If a test with high Sp cannot be used, one could use a less efficient test twice at recruitment or for identifying incident cases and with a serial interpretation. The loss *in Se* of such an approach would cause little bias, compared to the potential gains due to the increased Sp. However, this combined misclassification would also require a highly sensitive test to estimate an association close to the true RR. Unfortunately increasing Sp of a test very often decreases its Se, i.e., a lower probability for diseased individuals to be recognized as diseased. As a results, some classification errors are to be expected leading to biased parameters estimates. If classification errors cannot be avoided during the study design stage, the misclassification bias can be corrected into the analytic stage. For instance, Se and Sp of the test can be incorporated into the modelling strategy (35), by performing a probabilistic sensitivity analysis (36), or by including the uncertainty in the estimates with a Bayesian analysis in the form of prior distributions (37). A latent class model (38) would therefore return the posterior inference on regression parameters and the Se and Sp of both tests. Acknowledgement of these biases and possible corrective measures are important when designing longitudinal studies when gold standard measurement of the outcome might not be readily available, like for bacterial diseases (for example subclinical intramammary infection (39), viral diseases (40) or more complex outcome evaluations (e.g., bovine respiratory disease complex (41). Efforts should be made to improve outcome evaluation but absence or limitation of bias is not always granted in some situation (34). demonstrated that for some specific disease incidences and prevalences bias could not be avoided by improving outcome measurements. Using latent class models can help in these cases, as shown by (42).

Bias in parameters estimates can be important when considering selection and misclassification biases together in a cohort study. Our results underscore the need for a careful evaluation of the best available options to identify at risk and incident cases according to the expected disease prevalence and incidence of the study.

## Author Contributions

DH conducted the simulations, data analysis, results interpretation, and the manuscript writing. ID and SD contributed in interpreting the results and editing the manuscript. DH, ID, and SD contributed to the planning of the study.

## Conflict of Interest Statement

The authors declare that the research was conducted in the absence of any commercial or financial relationships that could be construed as a potential conflict of interest.
