# Supplementary material for: Selection and Misclassification Biases in Longitudinal Studies
Source: Front Vet Sci. 2018 May 28;5:99. doi: 10.3389/fvets.2018.00099 (PMC5985700; doi:10.3389/fvets.2018.00099)
Supplement: Supplementary file 1 [file Image1.PDF]

Supplementary Material:
Selection and Misclassification Biases in Longitudinal Studies

1 SUPPLEMENTARY FIGURES

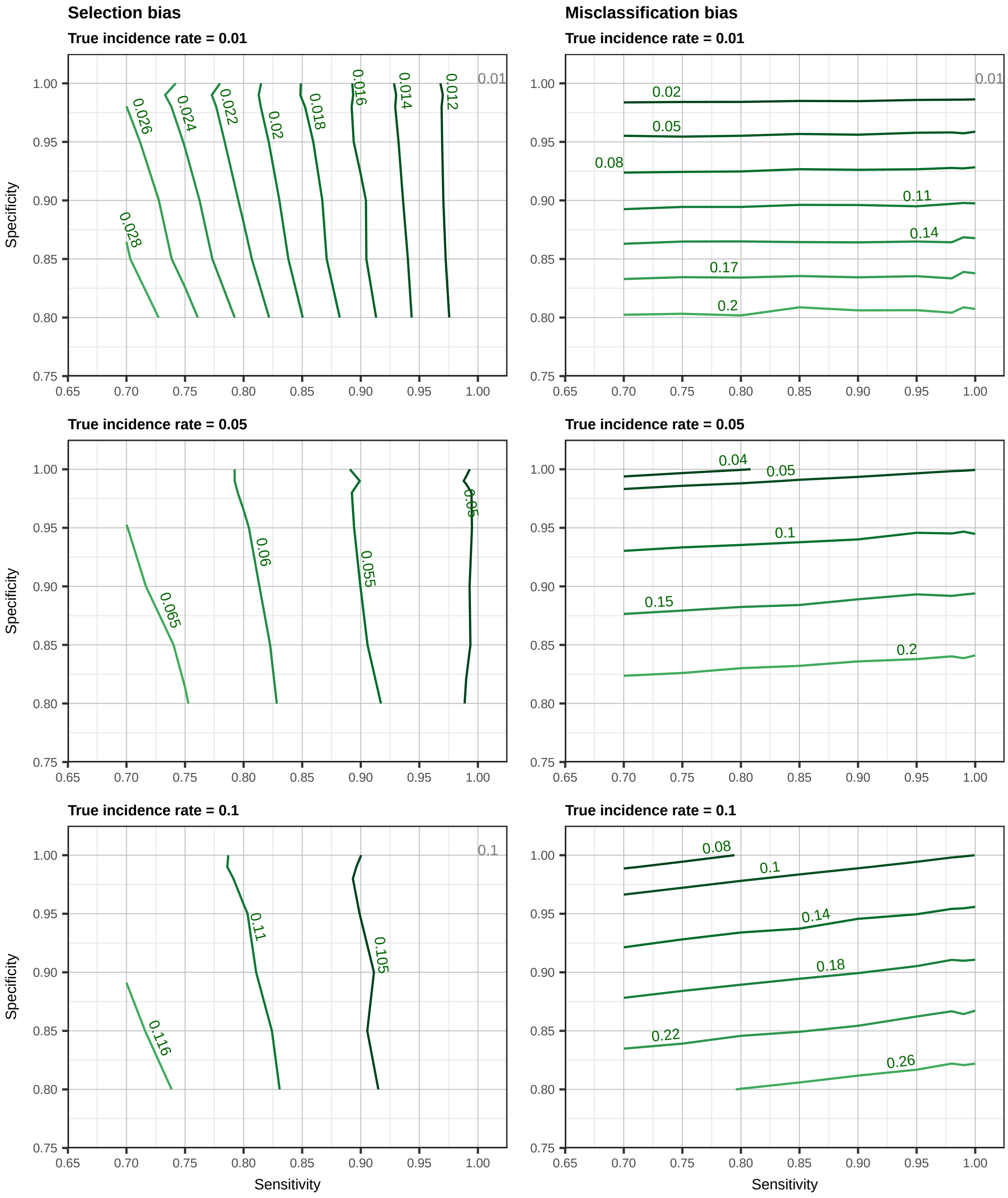

Figure S1: Estimated incidence rate as a function of test sensitivity and specificity, a disease prevalence of 5%, and true disease incidence (0.01, 0.05, 0.1 case/animal-time unit) when using an imperfect test at baseline (selection bias) or at follow-up (misclassification bias). True incidence rate is found at the upper right corner (i.e. perfect sensitivity and specificity).

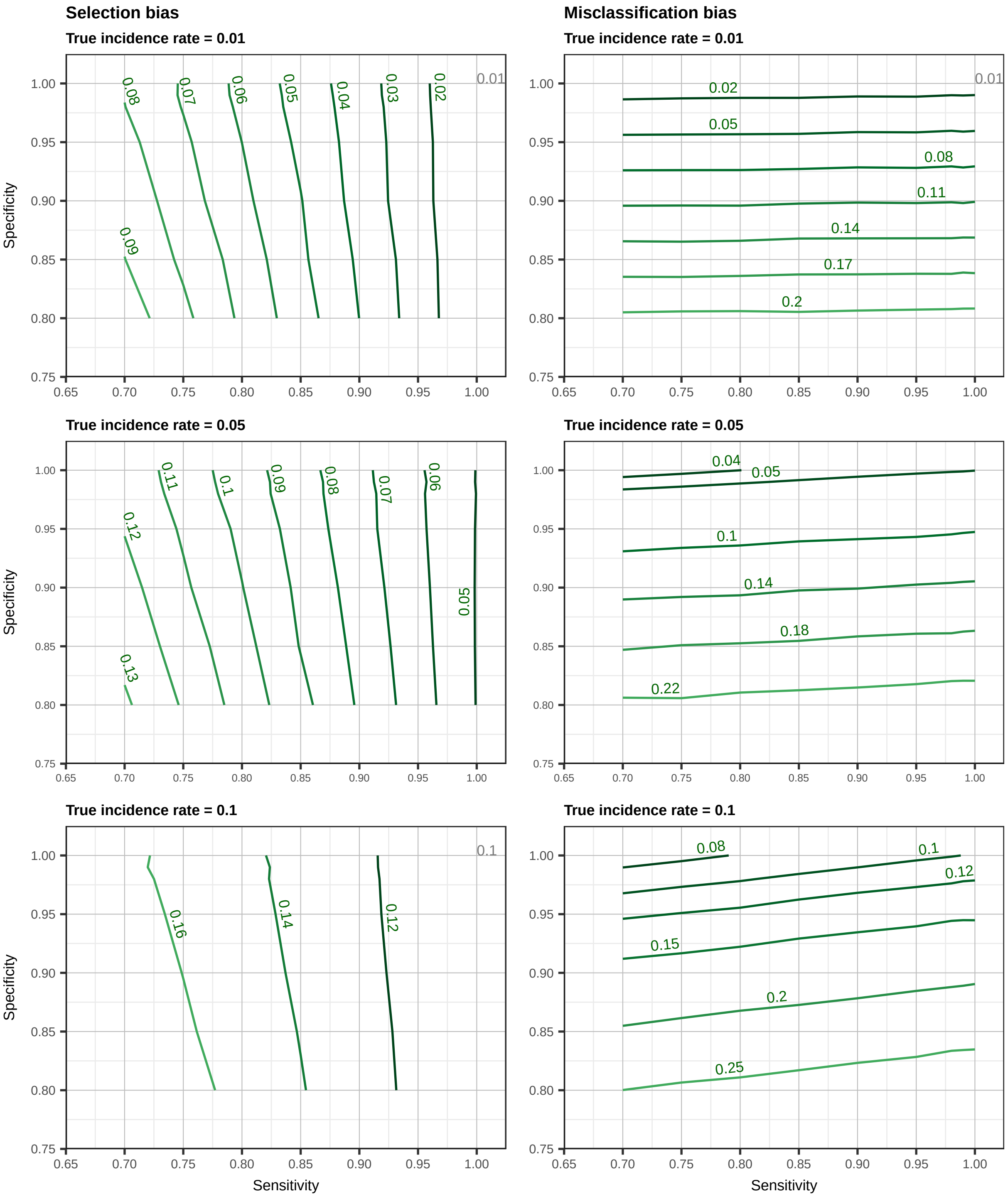

Figure S2: Estimated incidence rate as a function of test sensitivity and specificity, a disease prevalence of 20%, and true disease incidence (0.01, 0.05, 0.1 case/animal-time unit) when using an imperfect test at baseline (selection bias) or at follow-up (misclassification bias). True incidence rate is found at the upper right corner (i.e. perfect sensitivity and specificity).

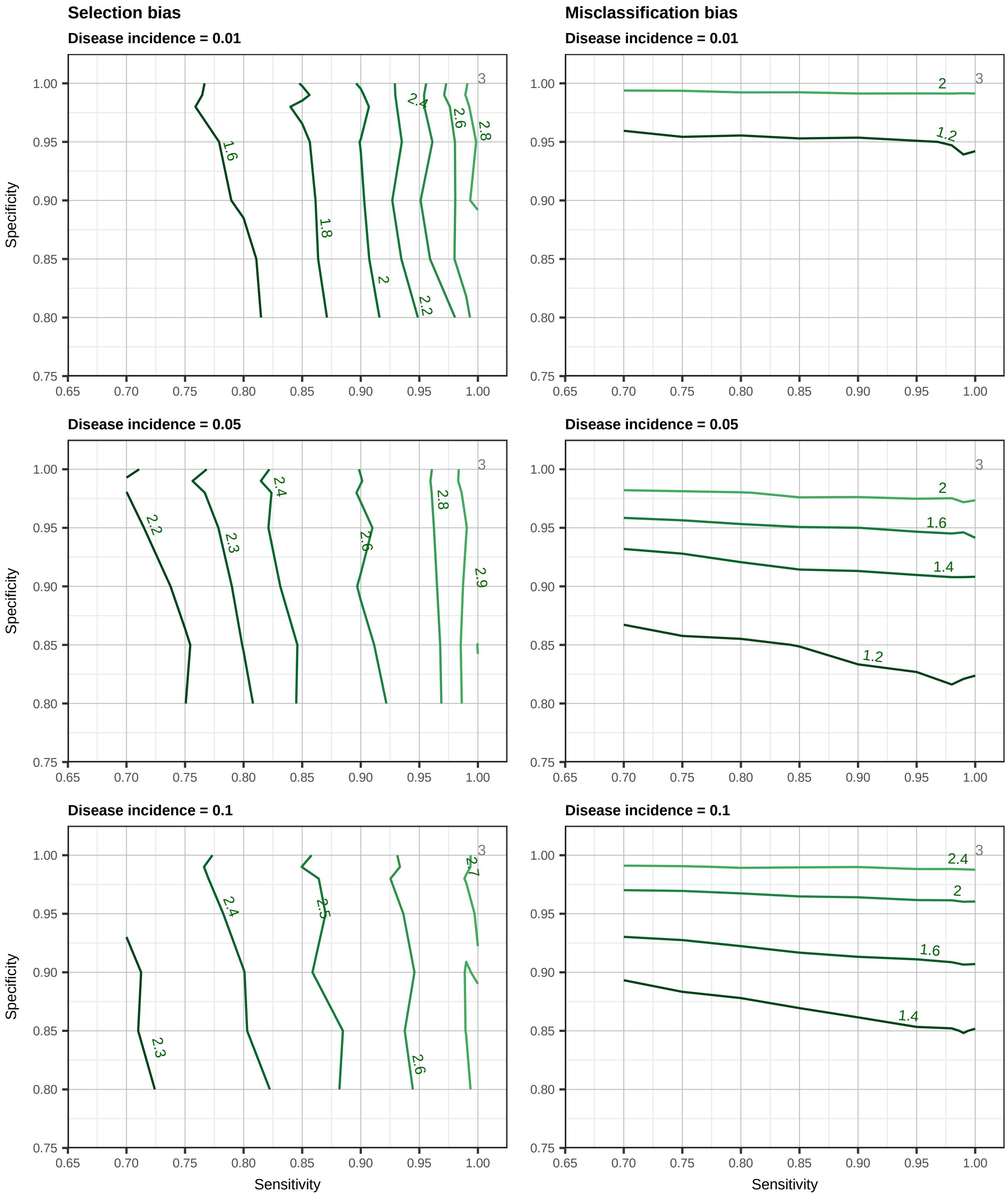

Figure S3: Estimated risk ratio as a function of test sensitivity and specificity, a disease prevalence of 5%, and true disease incidence (0.01, 0.05, 0.1 case/animal-time unit) for an exposure with a true measure of association corresponding to a risk ratio of 3.0 when using an imperfect test at baseline (selection bias) or at follow-up (misclassification bias). True risk ratio is found at the upper right corner (i.e. perfect sensitivity and specificity).

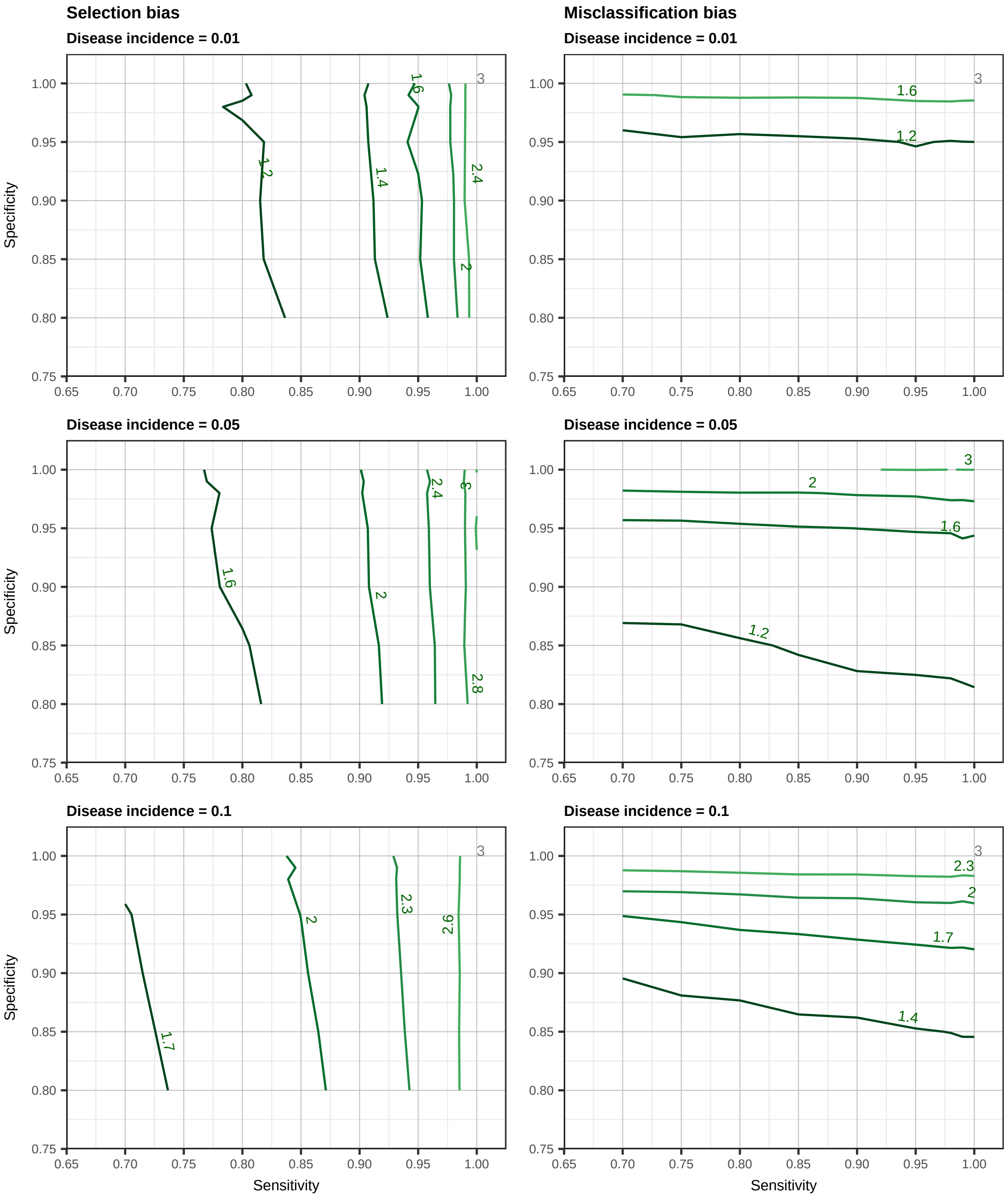

Figure S4: Estimated risk ratio as a function of test sensitivity and specificity, a disease prevalence of 20%, and true disease incidence (0.01, 0.05, 0.1 case/animal-time unit) for an exposure with a true measure of association corresponding to a risk ratio of 3.0 when using an imperfect test at baseline (selection bias) or at follow-up (misclassification bias). True risk ratio is found at the upper right corner (i.e. perfect sensitivity and specificity).

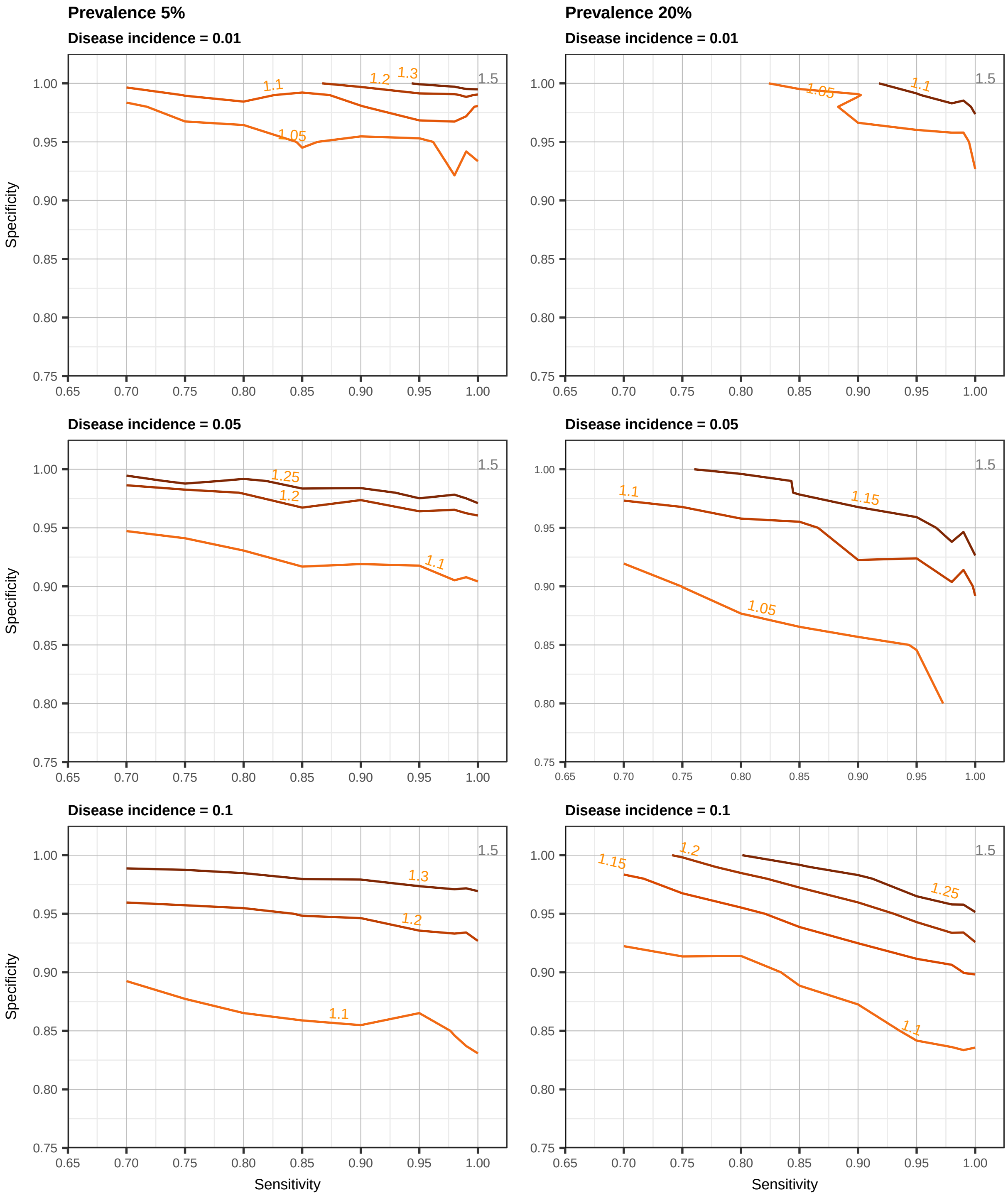

Figure S5: Estimated risk ratio as a function of test sensitivity and specificity, disease prevalence (5 or 20%), and true disease incidence (0.01, 0.05, 0.1 case/animal-time unit) for an exposure with a true measure of association corresponding to a risk ratio of 1.5 when using an imperfect test both at baseline and follow-up (i.e. total bias). True risk ratio is found at the upper right corner (i.e. perfect sensitivity and specificity).

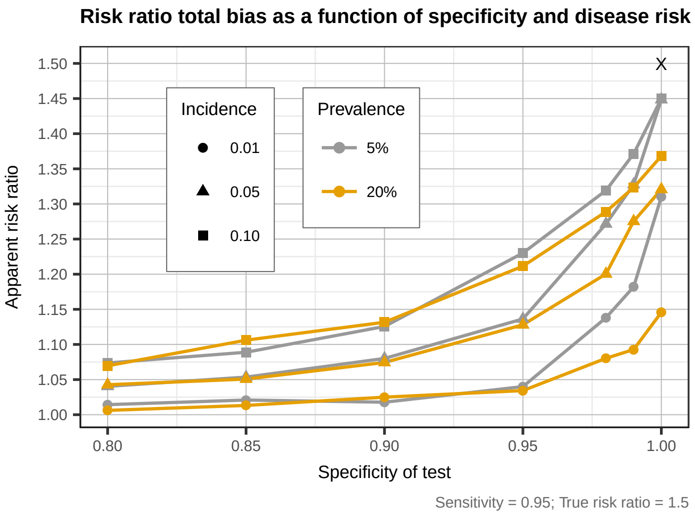

Figure S6: Estimated risk ratio as a function of test specificity and disease risk, and for a sensitivity of 95%, when using an imperfect test both at baseline and follow-up. True risk ratio = 1.5.

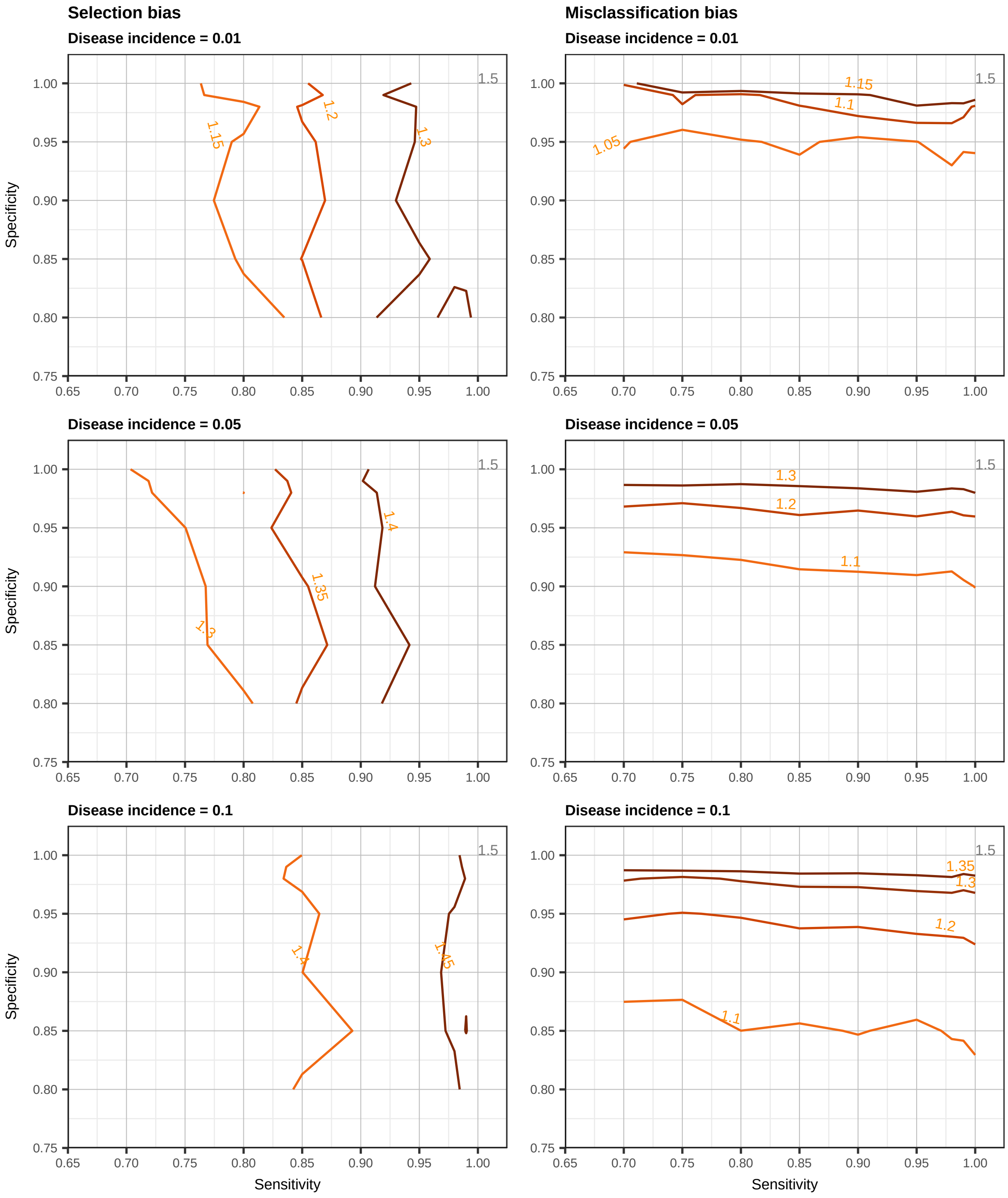

Figure S7: Estimated risk ratio as a function of test sensitivity and specificity, a disease prevalence of 5%, and true disease incidence (0.01, 0.05, 0.1 case/animal-time unit) for an exposure with a true measure of association corresponding to a risk ratio of 1.5 when using an imperfect test at baseline (selection bias) or at follow-up (misclassification bias). True risk ratio is found at the upper right corner (i.e. perfect sensitivity and specificity).

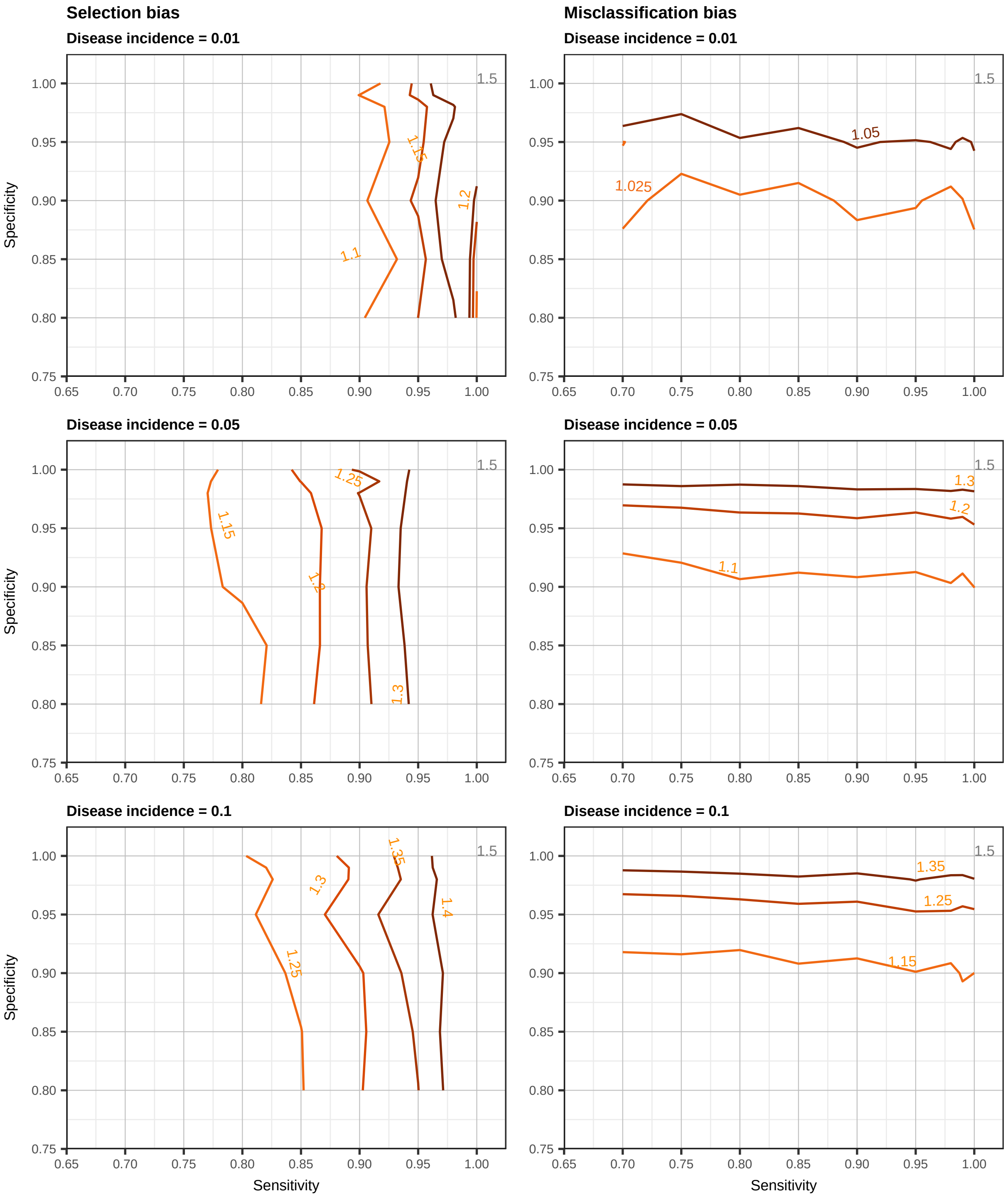

Figure S8: Estimated risk ratio as a function of test sensitivity and specificity, a disease prevalence of 20%, and true disease incidence (0.01, 0.05, 0.1 case/animal-time unit) for an exposure with a true measure of association corresponding to a risk ratio of 1.5 when using an imperfect test at baseline (selection bias) or at follow-up (misclassification bias). True risk ratio is found at the upper right corner (i.e. perfect sensitivity and specificity).
